# Is predation by ants driving the evolution of tarantulas? First report of tarantulas (Araneae, Theraphosidae) actively using urticating setae against invertebrates

**DOI:** 10.1038/s41598-026-64167-7

**Published:** 2026-08-03

**Authors:** Rogério Bertani

**Affiliations:** Laboratório de Ecologia e Evolução, Instituto Butantan, Avenida Vital Brazil, 1500, São Paulo, 05503-900 Brazil

**Keywords:** Army ant, *Camponotus rufipes*, *Eciton burchelli*, Defensive behavior, Prey‒predator evolution, Ecology, Ecology, Evolution, Zoology

## Abstract

The use of urticating setae by tarantulas from the Americas and the Caribbean has been known for centuries and is considered a defensive behavior against vertebrate predators. However, their passive use against invertebrates, such as parasitic fly maggots and ants, has also been recently proposed, but no active use of these setae against invertebrates has been observed or tested. In this study, eight tarantula specimens representing four different species from the family Theraphosidae were tested in twelve tests with the army ant *Eciton burchelli* and the carpenter ant *Camponotus rufipes*. Four spiders interacted with *Eciton burchelli*, and one interacted with *Camponotus rufipes*. The defensive behavior was distinct from that used against vertebrates, as the spider rubs the lower posterolateral region of the abdomen with downward and outward movements of the hind legs and uses silk threads. Scanning electron microscopy (SEM) demonstrated that the ants were entangled with urticating setae, primarily of type I morphology. Type I urticating setae are found exclusively in an important clade of theraphosine spiders, the tribe Theraphosini, which includes the largest known spiders. It is hypothesized here that protection against predation by ants due to the emergence of a specific type of urticating seta may be one of the factors responsible for the success of tarantulas in the New World.

**Supplementary Information:**

The online version contains supplementary material available at 10.1038/s41598-026-64167-7.

## Introduction

Several species of tarantulas are among the largest and longest-lived terrestrial invertebrates and are included in the family Theraphosidae. They are ubiquitous inhabitants of tropical and subtropical regions on most continents, with the exception of Antarctica^1,2^.

Species from the Americas and the Caribbean have developed an interesting defensive behavior of using urticating setae against potential predators and parasites, which has been recorded by naturalists since the 19th century^3,4^. When a tarantula is disturbed, it usually flicks the abdomen dorsum with its hind legs, dislodging the setae, which are carried by the air and can contact the skin and eyes of the aggressor^5^. They produce skin rashes and intense itching and may eventually cause damage to the human eyes^5,6^.

**Fig. 1:**
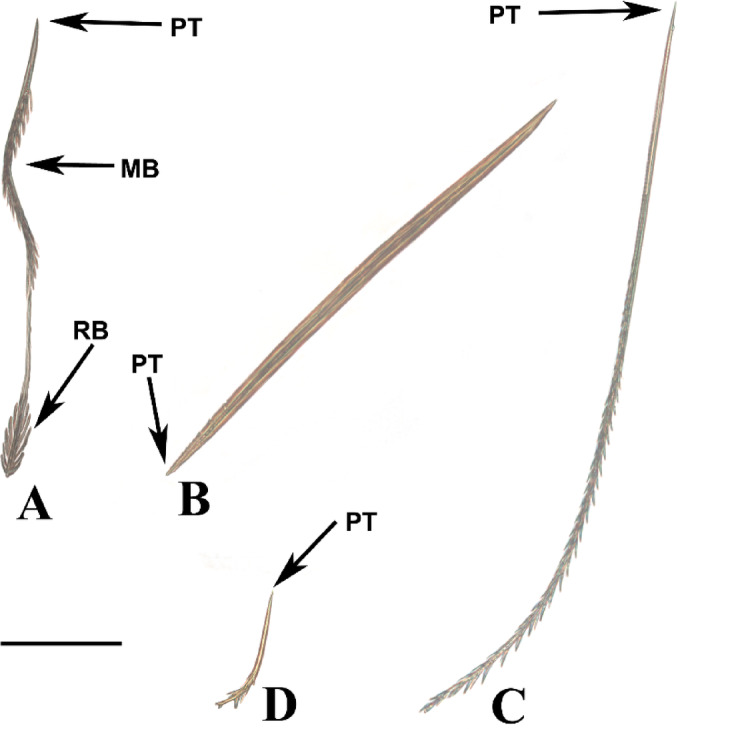
Main morphological types of urticating setae of Theraphosidae, according to Cooke et al.^5^ (**A**) Type (I) (**B**) Type (II) (**C**) Type (III) (**D**) Type (IV) Scale bar = 0.1 mm. MB = main barbs; PT = penetrating tip; RB = reversed barbs.

The morphology of some of these setae was first documented by Bertkau^7^, but only studied in any detail almost a century later^5^. Cooke et al.^5^ classified urticating setae into four types (I, II, III, IV) according to their morphology (Fig. 1A-D). Types I, III and IV are found on the abdomen dorsum of species now included in Theraphosinae, the largest Theraphosidae subfamily with 628 species and endemic to the Americas and the Caribbean^1^. Urticating setae of types I and/or III are long, slender and have well-developed barbs (Figs. 1A, C). They are attached to the spider’s abdomen via a short stalk, and the penetrating tip lies at its distal portion. Type III setae (Fig. 1C) are typically the longest and most slender urticating setae, ranging from 0.3 to 1.8 mm in length. Type IV setae are very short (0.06 to 0.2 mm in length) (Fig. 1D), and their use is not well understood (but see Russi & Pérez-Miles^8^. Type I ranges from 0.2 to 0.6 mm in size and is unique because of its morphology. There is an area of barbs directed toward its base, or the “main barbs”, followed by a region lacking well-developed barbs and then a region of “reversed barbs” close to its base^5^(Fig. 1A). Type II setae (Fig. 1B) were considered to be exclusive to the genus *Avicularia*^5^; however, more recently, this seta was found to be present in almost all 7 genera and 32 species of Aviculariinae, a subfamily composed of arboreal species distributed exclusively in the Americas and the Caribbean^1,9,10^. Type II intrigued Cooke et al.^5^ due to its shape, which is thicker than those of the other types, almost entirelly covered with weakly developed barbs, and the position of the penetrating tip is in a basal position (Fig. 1B). A few decades later, these setae were shown to be used in direct contact with potential predators when the spider rubs the abdomen dorsum against the aggressor^11^. Type V was described by Marshall & Uetz^12^ and is exclusive to the genus *Ephebopus*, with five species distributed in northern South America and belonging to the small subfamily Psalmopoeinae. Interestingly, the urticating setae are located in the distal prolateral area of the femur palp and are released by movements of the palps against the chelicerae (see 1 and 2 in Marshall & Uetz^12^. Two other morphological types have been described as types VI and VII for the theraphosine genera *Hemirrhagus* (see 4 in Pérez-Miles^13^ and *Kankuamo* (see fig. 2 in Perafán et al.^14^) from Mexico and Colombia, respectively^13,14^. Type VI is inserted directly into the stalks with penetrating tips^13^ and type VII morphologically resembles type II, but the penetrating tip is distal^14^. Table 1 summarizes information about the types of urticating setae.

**Fig. 2:**
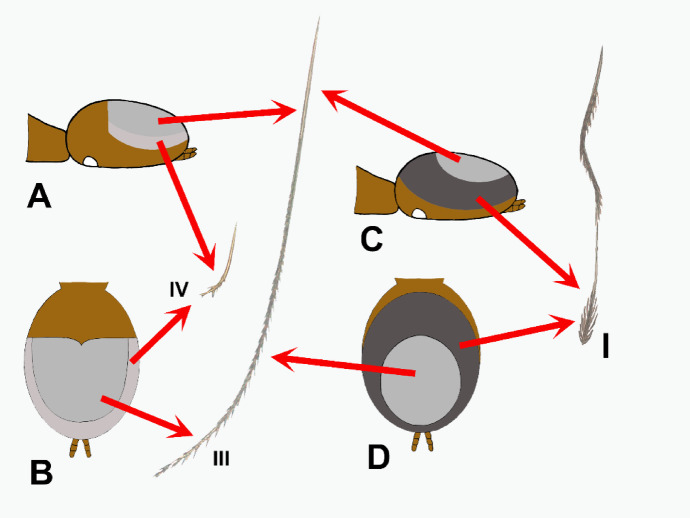
Distribution of urticating setae of types I, III and IV on the abdomen of representative theraphosine species, based on Bertani & Guadanucci^15^. AB *Grammostola* sp., (**A**) Abdomen, lateral. (**B**) Abdomen, dorsal. CD *Lasiodora* sp. (**C**) Abdomen lateral. (**D**) Abdomen dorsal. When occurring together with other types, type III urticating setae are always located in the median and median posterior regions of the abdomen, surrounded by type I or type IV urticating setae.

**Fig. 3:**
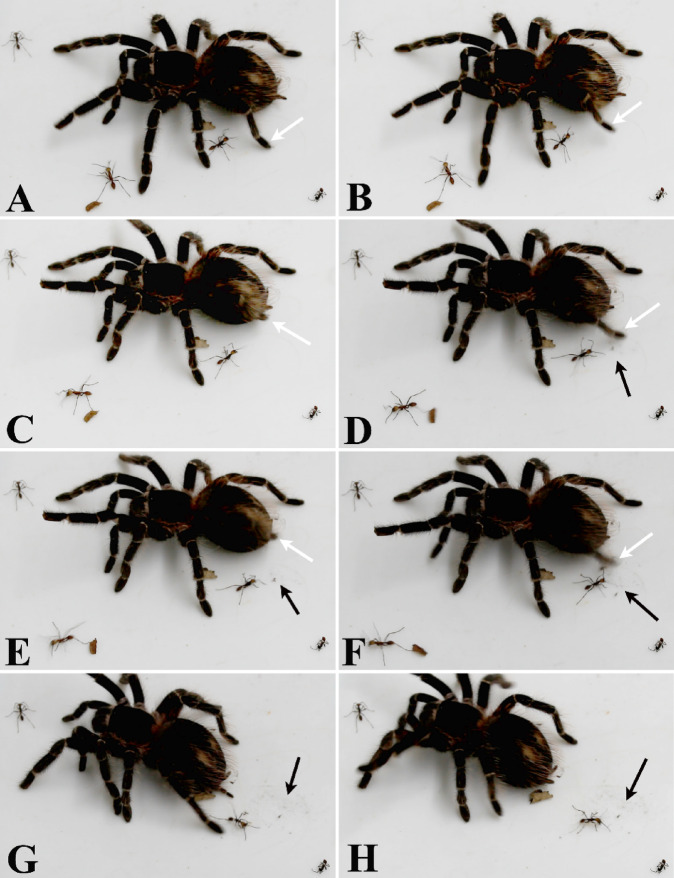
Defensive behavior of a tarantula (*Lasiodora parahybana*) using urticating setae against the army ant *Eciton burchelli*. (**A**) The spider is resting while army ants are moving around it. An ant approaches the spider between its left third and fourth legs (white arrow). (**B**, **C**, **D**) First movements of the left leg IV (white arrows), raising the leg (**B**), making contact with the lower posterolateral region of the abdomen (**C**) and throwing urticating setae against the substrate (black arrow) near a passing army ant. (**E**, **F**, **G**) Second movements of the left leg IV, again raising the leg, rubbing the abdomen (E) and releasing urticating setae and silk threads (**F**). (**G**, ** H**) The spider moves away while the ant becomes entangled by urticating setae and silk threads.

**Fig. 4:**
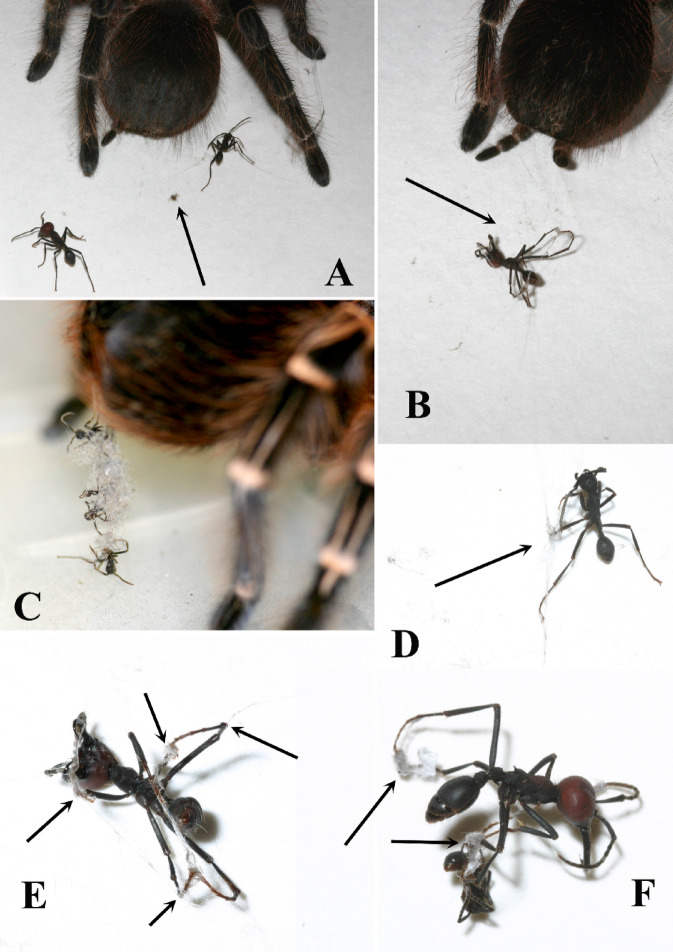
Defensive behavior of tarantulas using urticating setae against the army ant *Eciton burchelli*. (**A)** An army ant can be seen entangled in silk threads and urticating setae near the spider *Lasiodora benedeni* (above|), while another ant moves below. A cluster of type I urticating setae can be seen nearby (black arrow). Due to their morphology, type I urticating setae become entangled, forming clusters of setae that are difficult to carry through the air and tend to fall to the ground. An ant’s contact with a cluster of type I setae can release and entangle hundreds of setae with the ant’s appendages (**B**) An army ant is seen entangled in silk threads and urticating setae. The black arrow indicates a layer of silk and urticating setae near the ant. (**C**) Four army ants entangled in silk threads and urticating setae are attached to the spinnerets of the tarantula *Acanthoscurria geniculata*. (**D**, **E**, **F**) Army ants entangled in silk threads and urticating setae (arrows). In (**F**), two ants became entangled with each other and died.

Cooke et al. (1972)^5^ showed that type III can occur together with types I or IV in the same specimen, but it was not known whether these setae were intermixed or segregated in distinct areas on the spider’s abdomen. More recently, it has been demonstrated that urticating setae of types I and III or III and IV, when they occur together, are found in distinct areas of the spider’s abdomen^15^. Type III setae are always located in the central and posterior areas of the abdomen and are surrounded by type I or IV setae (Fig. 2).

Cooke et al.^5^ also tested the behavior of some potential mammalian predators from the United States when in contact with local tarantulas of the genus *Aphonopelma*. They reported that the tested predators exhibited severe discomfort after contact with the urticating setae released by the spiders. The tarantula species tested by Cooke et al.^5^ with predators and humans possess urticating setae of types I or I and III. Cooke et al.^5^ emphasized that type III setae are much more urticating than type I setae and penetrate deeply into the skin.

The use of urticating setae in a defensive way against vertebrates, mainly mammals, was well established, but nothing was known about their use against invertebrates until Marshall & Uetz^16^. Unlike active use, i.e., stimulated by the presence of a potential predator, the use of urticating setae reported by Marshall & Uetz^16^ was passive, i.e., independent of the presence of a predator and used to prevent possible predation or parasitism. They demonstrated that the tarantula *Theraphosa blondi* can incorporate type III urticating setae into the molting webs, which are produced by the spider to cover the area where it will undergo ecdysis, and into the egg sac wall. Egg sacs and molting webs of *Theraphosa blondi* were also tested with dipteran maggots of *Megaselia scalaris* (Phoridae), a well-known scavenger. They reported that the movements of maggots were slowed by the setae incorporated into the silk, whereas molting webs lacking incorporated urticating setae were less effective. Their experiments were carried out with *Theraphosa blondi*, a species that carries only type III urticating setae of two different lengths. They concluded that incorporating urticating setae into the silk layer of molting webs and egg sacs would be an effective protection against parasitism by scavenger fly maggots^16^.

Marshall & Uetz also observed a female *Megaphobema* sp. incorporating urticating setae into the egg sac originating from the anterior lateral areas of the abdomen^16^.This area is composed mainly of type I urticating setae (Figs. 1 and 2), but the authors did not mention the type or urticating setae present in the egg sac. They also tested the response to contact of the egg sac with humans and two species of mice (*Mus musculus* and *Peromyscus* sp.) which did not show an urticarial effect^16^.

In another more recent publication^15^ tests with phorid fly maggots and ants (*Camponotus rufipes*) in molting webs with urticating setae showed that type I setae are more efficient in deterring these potential parasites and predators owing to their morphology, which has an area of reversed barbs that aids in containing the movements of the maggots and in entangling the ants^15^ .

The greatest diversity of tarantula species is concentrated in the Americas and the Caribbean, with 815 (68.37%) of the 1192 species described worldwide. They can be found from the central United States in North America to central Argentina and Chile in South America and on most Caribbean islands^1^. Urticating setae arose convergently at least three times during theraphosid evolution^15,17,18^, all in the Americas and the Caribbean: in Psalmopoeinae (5 species), Aviculariinae (33 species) and Theraphosinae (Table 1). Therefore, the presence of urticating setae, regardless of their morphological type, appears to represent an evolutionary advantage. Among these, Theraphosinae is the most species-rich subfamily, with 625 species, representing 76.68% of the tarantula species in the Americas and the Caribbean and 52.4% of the world’s species. One of the synapomorphies of Theraphosinae is the presence of urticating setae of types I, III, and IV, which may occur individually or together, such as I and III or III and IV, in the same specimen^5,15^ (Figs. 1 and 2).

Biswas et al.^18^ used tarantulas as a model to investigate the relative influence of clade age and diversification rate variation hypotheses on the asymmetry of species richness distribution among the theraphosid subfamilies. They chose tarantulas because they are a speciose group of spiders distributed worldwide, but exceptionally diverse in South America. Using trait-independent and dependent diversification models, they tested “the clade age hypothesis, the role of the microhabitat, antipredator defense strategy, and geography in influencig diversification rates”. They found that clade age is the main predictor of species richness distribution among tarantula subfamilies, explaining 40% of the variation^18^. However, their results sugested that, in Theraphosinae, “the presence of urticating seta probably disrupted this pattern in some clades by increasing the net diversification rates, not by increasing the speciation rate but by reducing the extinction rate”^18^. As tarantulas are the only group of arachnids with urticating setae, “tarantulas appear to have had a unique evolutionary trajectory where the gain of novel defensive traits has increased diversification rates, most likely by reducing extinction rates rather than increasing speciation rates supporting the “escape-and-radiate” hypothesis”^18^.

If the presence of urticating setae may have had a primary role in the evolution of tarantulas of the Americas and the Caribbean, it is necessary to understand which predators and selective pressures are acting. Vertebrates are possibly among the main predators of tarantulas, and the use of type III urticating setae may confer protection against predation by these animals. However, little is known about the use of type I urticating setae, present in 335 (53.34%) theraphosine species, representing 41.1% of tarantulas in the Americas and the Caribbean. In addition to its unusual morphology, type I urticating setae have been shown to be weakly urticating to vertebrates^5^. Nevertheless, It was shown that this type of seta can be passively effective against at least one species of ant when incorporated into a molting web^15^.

Zamani et al.^19^ proposed that hirsuteness in theraphosid spiders, mainly in Theraphosinae, may have evolved in part as a defensive strategy against army ants, since some physical defensive barriers are well known in insects that are effective in protecting against ant predation. They also presented a series of evidence on the basis of natural history observations, in which army ants swarmed over tarantulas without aggression from the ants or reactions from the spider.

Ants are among the most abundant and species-diverse invertebrate predators in the tropics and subtropics^20^. In particular, army ants can cause a drastic decrease in leaf-litter arthropods^20–22^, which can occur through direct predation by ants or by subsequent predation or parasitization by swarm followers^23^. In the Central Amazon, predation by *Eciton burchelli* can have a very high impact on ground-living spider populations, mainly on median-sized species in the genus *Ctenus* (Ctenidae) and on mygalomorphs, which are important prey of ants, whereas the other sympatric ant, *Labidus praedator*, feeds on smaller individuals of other spider species^22^. Therefore, ants, especially army ants, can be considered potential predators of tarantulas that live in tropical and subtropical terrestrial habitats, typically in areas where swarms of these ants forage.

To date, only reports of the potential passive use of urticating setae against invertebrates are known^15,16^. In this study, I tested two potential invertebrate predators—the army ant *Eciton burchelli* and the carpenter ant *Camponotus rufipes*—with some theraphosid spiders, which exhibited an active defensive display using urticating setae toward the ants. The role of specific urticating setae types in preventing predation by ants and their potential evolutionary advantages are also discussed.

## Results

### Defensive behavior tests

**Fig. 5:**
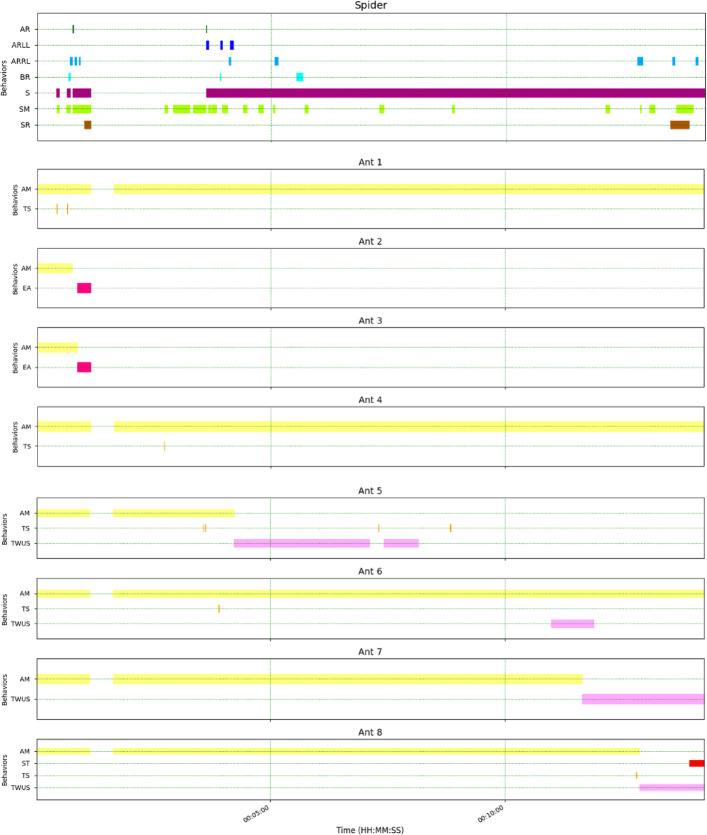
Ethogram of recorded behaviors of the representative specimen Lb. AM = ant moving; AR = Tarantula abdomen raising; ARLL = Tarantula abdomen rubbing, left leg; ARRL = Tarantula abdomen rubbing, right leg; BR = Tarantula body rotation; EA = Ant entangled with another ant; S = Tarantula spinning; SM = Tarantula moving; SR = Tarantula silk raising; ST = Ant struggling; TS = Ant touching the tarantula; TWUS = Ant trapped in the web with urticating setae.

In the first experiment (2004), *Eciton burchelli* were tested (Figs. 3A-H, 4A-F and 5; Tables 2  and 3, Supplementary Movie 1).

**Table 1:** Types of urticating setae, location on spider body’s, presence on taxa and number of species.

Urticating setae type Location Taxa			Number of species
I^1 ^	Abdomen	Theraphosinae	335
II^1^	Abdomen	Aviculariinae	33
III^1^	Abdomen	Theraphosinae	262
IV^1^	Abdomen	Theraphosinae	
V^2^	Palps	*Ephebopus* (Psalmopoeinae)	5
VI^3^	Abdomen	*Hemirrhagus* (Theraphosinae)	27
VII^4^	Abdomen	*Kankuamo* (Theraphosinae)	1

After ^1^Cooke et al.^5^; ^2^Marshall & Uetz^12^; ^3^Pérez-Miles^13^; ^4^Perafán et al.^14^

**Table 2:** Specimens and species tested with the ants *Eciton* and *Camponotus*.

Specimen	Species	Locality	Biome	*Eciton*	*Camponotus*
Ag1	*Acanthoscurria geniculata*	Xingú, State of Pará, Brazil	Amazon Forest	P	
Ag2	*Acanthoscurria geniculata*	Medicilândia, State of Pará, Brazil	Amazon Forest	N	N
Lp1	*Lasiodora parahybana*	Baia Formosa, State of Rio Grande do Norte, Brazil	Brazilian Atlantic Forest	P	N
Lp2	*Lasiodora parahybana*	Baia Formosa, State of Rio Grande do Norte, Brazil	Brazilian Atlantic Forest	P	N
Lp3	*Lasiodora parahybana*	Rio Formoso, State of Pernambuco, Brazil)	Brazilian Atlantic Forest		N
Lp4	*Lasiodora parahybana*	Born in captivity, mother from Murici, State of Alagoas, Brazil	Brazilian Atlantic Forest		P
Lk	*Lasiodora klugi*	Ilhéus, State of Bahia, Brazil	Brazilian Atlantic Forest	N	N
Lb	*Lasiodora benedeni*	Taubaté, State of São Paulo, Brazil	Brazilian Atlantic Forest	P	

P = positive, there were interactions between the spider and the ant. N = negative, no interaction was recorded.

**Table 3:** Summary of recorded behaviors for the tarantulas and ants tested.

Behavior	Records				
* Tarantulas*	Ag1 (E)	Lb (E)	Lp1 (E)	Lp2 (E)	Lp4 (C)*
Abdomen raising	2	2	0	0	2
Abdomen tilting	1	0	0	0	1
Silk raising	1	2	1	1	0
Spinning	1	4	2	1	1
Abdomen rubbing, left leg	1	3	1	1	0
Abdomen rubbing, right leg	3	8	1	1	2
Body rotation	0	3	0	0	0
*Ants*					
Touching the tarantula	2	9	7	0	1
Entangled to another ant	5	3	1	3	0
Trapped in the web	5	5	2	3	0
Struggling	5	1	2	3	0

E = *Eciton*. C = *Camponotus*.

Non-interactive behaviors (resting, moving) were not quantified here. *The specimen released few urticating setae, therefore the effect on the ants was minimum.

Ag1, in a plastic box. A few minutes after the ants were released, the spider slightly raised its abdomen, tilting it toward the approaching ants while spinning a layer of silk. The spider then rubbed the distal part of the metatarsus IV against the lower posterolateral area of the abdomen sixteen times in approximately three seconds with short downward movements followed by outward movements, releasing urticating setae onto a layer of silk. Three passing ants became trapped in the mixture of silk and urticating setae. As the silk remained fixed in the spider’s spinnerets, the combination of the web, urticating setae, and ants remained attached to the spinnerets and were suspended in the air (Fig. 4C). The spider moved while carrying the silk with the ants attached to the spinnerets.

After approximately nine minutes, the other ants approached the spider. Again, it slightly raised its abdomen, directed it toward the ants, and rubbed the lower posterolateral area of its abdomen with the distal metatarsus IV, making eleven short downward movements followed by outward movements of leg IV in approximately three seconds. A new ant joined the other three ants attached to the spinnerets.

After two and a half minutes, another ant approached the spider, but from the other side. The spider tilted its abdomen to the other side, toward the ant, and, using its other leg IV, rubbed the lower posterolateral area of its abdomen fifteen times in approximately five seconds with its metatarsus, with the same downward and outward movements, releasing urticating setae onto the layer of silk.

Lb, in a plastic box (Figs. 4A, B and 5; Tables 2 and 3).

The spider was positioned head down on the wall of the box when the ten ants were released. When the ants approached, the spider made movements with its spinnerets and moved its abdomen laterally downward toward the ants. The spinnerets touched the substrate and released a silk sheet that was subsequently covered with urticating setae by rubbing metatarsus IV against the lower posterolateral area of the abdomen with thirteen short downward and outward movements in approximately four seconds. Then, it moved its abdomen, directing the posterior part downward toward another approaching ant, and repeated the above behavior with eight leg movements in approximately two seconds. Two ants became entangled with the silk and setae on their legs and had difficulty moving freely. After touching each other, the two ants become entangled themselves, with enormous difficulty in locomotion.

The same spider was placed inside an acrylic box with more *Eciton* ants. After two minutes, while moving, it made contact with a passing ant. The spider then touched the substrate with its spinnerets, releasing silk threads, and rubbed the lower posterolateral area of its abdomen with metatarsus IV seven times in approximately three seconds, again with short downward and outward movements, releasing urticating setae that were incorporated into the layer of silk and the ant. The substrate was covered by a layer of silk and urticating setae. It moved slowly and made contact with another ant after fifteen seconds, repeating the same behavior with six leg movements in approximately three seconds. After ten seconds, it repeated the movements after another contact with another ant. However, now it used both legs in six seconds, first the right leg in three movements, followed by the left leg in fourteen movements. The movements and areas of the abdomen touched by the spider were the same as those described previously. It moved slowly again, and after one minute, the left side of its abdomen was rubbed with its left leg twelve times in approximately four seconds. This time, there were no ants near the spider, and many urticating setae could be seen on the ground. The spider then began to cover the area with the setae using silk strands, paused for a few seconds, and began to weave a web around itself, covering the space between itself and the ants. Some ants became trapped in the silk with urticating setae. After some time, it was possible to see the ants trapped in the silk, which was fixed in the spinnerets and suspended in the air, similar to the previous experiment with Ag1.

In the second experiment (2025), *Eciton burchelli* was tested:

Ag 2 and Lk were tested in a plastic tray, and after fifteen minutes, there was no interaction with the ants.

Lp1 was tested on the same plastic tray used previously (Tables 2 and 3). The spider raised its spinnerets when an ant approached, probably releasing silk, and rubbed metatarsus IV fourteen times in approximately three seconds against the lower posterolateral area of its abdomen, releasing urticating setae on the layer of silk and the ant. The spider then moved while the ant became entangled in the silk and urticating setae, exhibiting erratic movements. The ant then touched another ant and became entangled with it.

Lp2, tested in the same plastic tray (Tables 2 and 3). The spider used its hind legs, moving and rubbing metatarsus IV five times in approximately a second against the lower posterolateral area of the abdomen while releasing silk threads against the approaching ants, as detailed above. Shortly afterward, the right metatarsus IV rubbed the abdomen in fifteen movements in approximately 3 s. Three ants became trapped in suspended silk threads fixed to the spider’s spinnerets.

In the second experiment (2025), *Camponotus rufipes* was tested:

Lp1, Lp2, Lp3, Lk, and Ag2 were tested in a plastic tray as described above, and after fifteen minutes, there was no interaction with the ants.

Lp4 (Tables 2 and 3), after contact with some ants, showed the same defensive behavior described above for *Eciton burchelli*, moving the right metatarsus IV fifteen times in approximately three seconds to rub the lower posterolateral area of the abdomen in the direction of the ant. The spider also performed movements with the spinnerets, touching them to the substrate. After 20 s, the spider repeated the same movements after another contact with an ant, with fifteen movements of the right leg in approximately three seconds.

### SEM findings

**Fig. 6:**
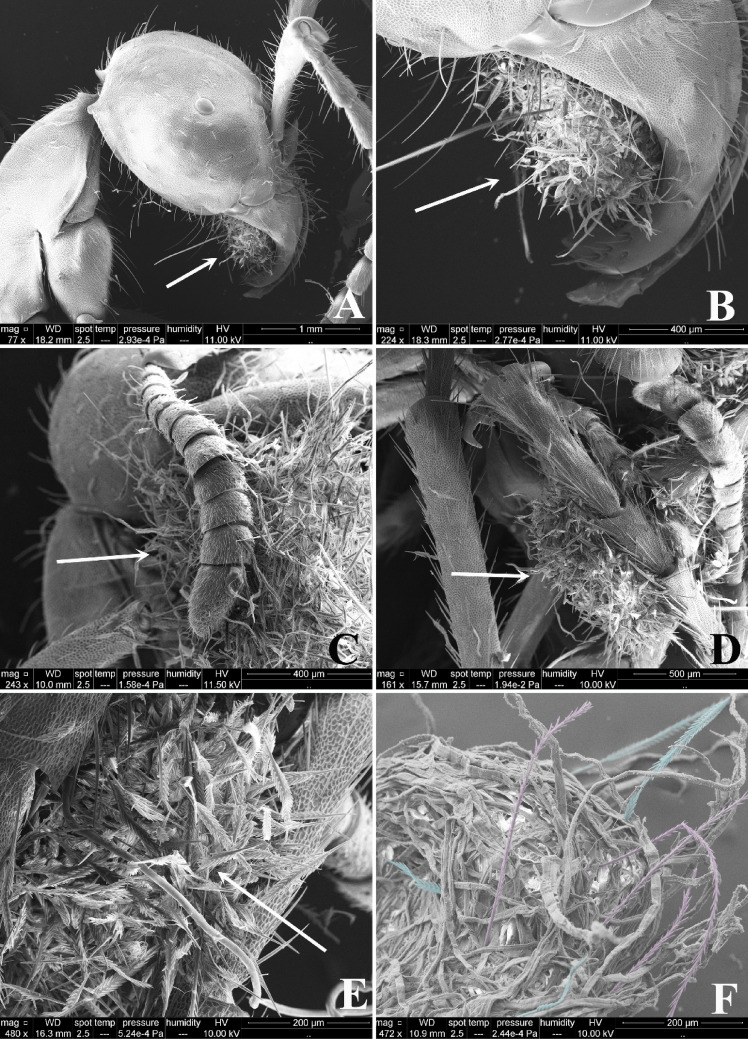
SEM micrographs of ants (*Eciton burchelli*) after contact with clusters of type I urticating setae. (**A**, **B**) A small cluster of type I urticating setae entangled in the ant’s mouthparts (arrows). (**C**, **D**, **E**) A large cluster of type I setae can be seen attached to the head (**C**) or legs (**D**, **E**) of the ant (arrows). (**F**) A cluster of mostly silk threads with sparse urticating setae of types I and III. Type I is artificially colored light blue, and type III is colored violet.

**Fig. 7:**
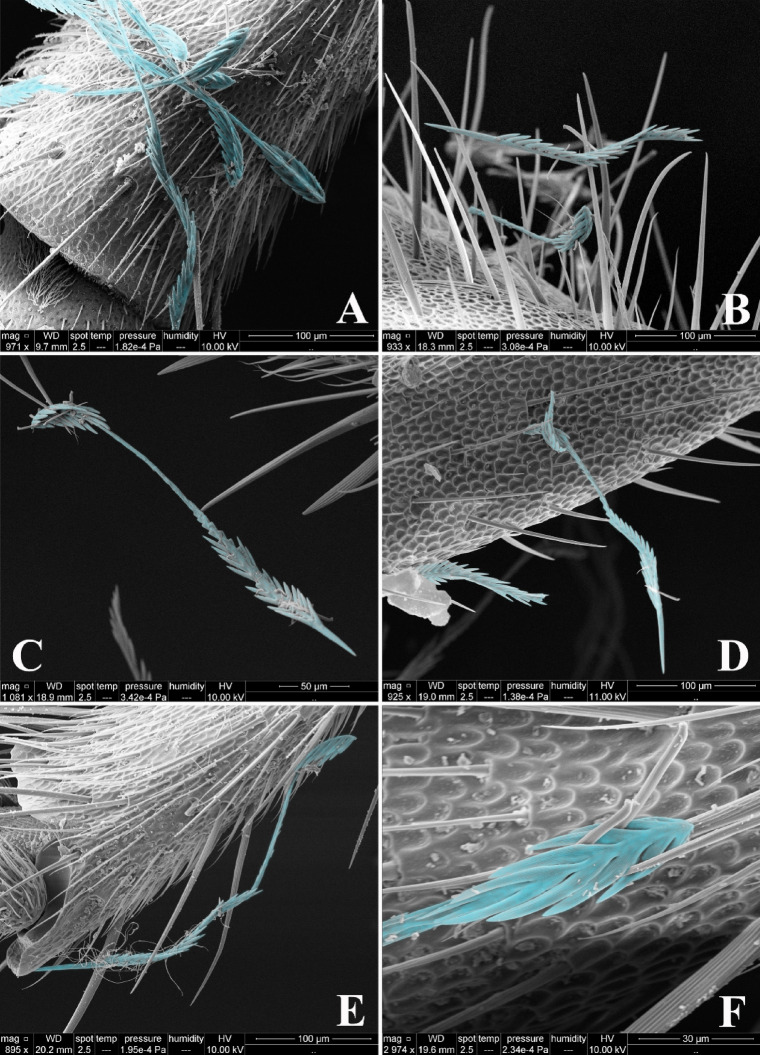
SEM micrographs of ants (*Eciton burchelli*) after contact with type I urticating setae. (**A**) The main barbs and reversed barbs of type I urticating setae intertwine with each other and with the setae covering the ant’s integument. (**B**) Broken type I seta showing the main barbed region (above) and the region of the reversed barbs (below) intertwined with the ant’s setae. Note that the type I seta has longer and broader barbs compared to the type III seta (Fig. 1C), allowing the ant’s seta to become entangled with it. (**C**) Type I urticating seta entangled in an ant seta by its reversed barbed region. (**D**) Type I urticating seta entangled in the broken reversed barbed region of another seta. (**E**) Type I urticating seta entangled by its main barbed region and reversed barbed region in the seta covering an ant’s leg. (**F**) Detail of the reversed barbed region of a type I urticating seta entangled around the seta covering an ant’s leg. Note that the thick barbs keep the ant’s setae attached to it. The type I urticating setae were artificially colored light blue to highlight their shape.

Examination of some tested ants using scanning electron microscopy (SEM) shows how the urticating setae of tarantulas act to prevent their movements. While some type III setae can be observed scattered (Fig. 6F), the setae entangled to the ants’ bodies are of type I (Figs. 6A-E and 7A-F). Type I urticating setae have thick, long barbs positioned close to each other, leaving a small space between them where the thin setae covering the appendages and body of the ants can become entangled (Fig. 7A-F). Figure 7F, in particular, shows a detailed image of this mechanism. Type I setae have two areas with barbs, oriented in opposite directions, the main barbed region and the reversed barbed region. Between these two areas there is an area without barbs, the length of which can vary. Thus, while one of the two areas becomes entangled in some setae on the ant’s body, the other part may become entangled in another part or seta of the same ant, in another ant, in other type I setae, or in silk threads deposited by the spider. In this way, the ant may suffer from difficulty moving to becoming completely immobilized (Fig. 4B-F).

Because type I urticating setae easily become entangled when released from the spider’s abdomen, they tend to form clusters that are not as easily carried out by the air as type III setae. They tend to remain on the ground and are very effective at hindering the movement of ants due to the huge number of urticating setae that come into contact with the ant (Fig. 6A-E, Supplementary Movie 1). When an ant is covered in urticating setae, it has difficulty moving and it is common for it to come into contact with another passing ant. The result is that the two (or more) ants become entangled and unable to move freely (Fig. 4F, Supplementary Movie 1). Attempts to escape and grooming to free themselves from the setae only cause them to become more entangled, as the setae interlock even more and can come into contact with other appendages, making escape more difficult.

Interestingly, notable cases of evolutionary convergence with tarantula type I urticating setae can be found in other invertebrates, both in terms of morphology and the use of urticating setae to deter ant attacks. Type I urticating setae closely resembles those found in polyxenid millipede and dermestid beetle larvae, as these defensive setae have thickened barbs and an area of reversed barbs^24–27^. The reversed barbed area of the “hastiseta” of the dermestid beetle larva has been termed “apical head”^24–26^ and “grappling hook” in *Polyxenus*^27^ and functions in the same way detailed above for the reversed barbs of type I urticating setae, entangling ants and hindering predation^24–27^.

## Discussion

The use of urticating setae against vertebrates has already been well established^5^ and the potential passive defensive use of tarantula urticating setae has been proposed^15,16^, but no active behavior using these setae against invertebrates has been reported. I tested the behavior of some tarantula species in direct contact with two large ant species, *Camponotus rufipes*, an omnivorous species widely distributed in South America, and the army ant *Eciton burchelli*, a carnivorous species distributed from southern North America to southern South America (Fig. 3A-H, Supplementary Movie 1)). Among the twelve tests performed, four tarantulas interacted with *Eciton burchelli*, and one interacted with *Camponotus rufipes* (Table 2). The interactions were similar for both ant species in terms of leg movements and the use of silk and urticating setae. This defensive behavior was distinct from that used against vertebrate predators, in which the spider uses its hind legs to rub the median and posterior regions of the abdomen^28^. In this case, the rubbed areas present mainly type III urticating setae (Figs. 1 and 2), which are long, thin, and easily carried by air and can disperse over a considerable distance, penetrating the respiratory tract and eyes of vertebrates^5^. Moreover, tarantulas do not use silk threads when kicking urticating setae against vertebrate predators. When the stimulus came from the ants, the movements of the hind legs were short, downward and outward, and the lower posterolateral area of the abdomen was the area touched, resulting in the setae being directed toward the ground (Fig. 2 A-H, Supplementary Movie 1). This abdominal area predominantly has type I urticating setae^15^ (Figs. 1 and 2C-D). Interestingly, the spider also used silk threads, which were deposited on the ground by the contact of the spinnerets, sometimes simultaneously with leg movements (Figs. 3A-H and 4A-F; Supplementary Movie 1). Silk threads, by themselves, could potentially hinder or partially prevent the movements of ants in a manner similar to that observed by Marshall & Uetz^16^ when webs lacking incorporated urticating setae of *Avicularia* with phorid fly maggots were tested. However, in this case, silk threads were used in conjunction with type I setae, which could potentially increase their effectiveness against ants.

Zamani et al.^19^ hypothesized that the hirsuteness in tarantulas could be a physical barrier against predation by ants. Nonetheless, some of the observations were made with arboreal tarantulas, which lack type I urticating setae and suffer less from ant predation, since most army ants, such as *Eciton burchelii*, forage in the leaf litter^20^. The only tarantula species reported by them as being preyed upon by *Eciton burchelli* was *Tapinauchenius plumipes*, an opportunistic species that lives on the ground or trees and lacks urticating setae. They also presented a few cases in which theraphosine species having type I setae came into contact with *Eciton burchelli* and were not attacked by the ants. As demonstrated by the results, tarantulas do not always react to ants, similar to how they do not always release urticating setae when stimulated by vertebrates (such as humans), preferring other forms of defense, such as escape, biting, immobilization, or physical barriers (hirsuteness). The hirsuteness of several tarantula species seems to act as a primary and general barrier against predation by ants, as suggested by Zamani et al.^19^, and the use of type I urticating setae would be another more specialized and effective defensive strategy.

**Fig. 8:**
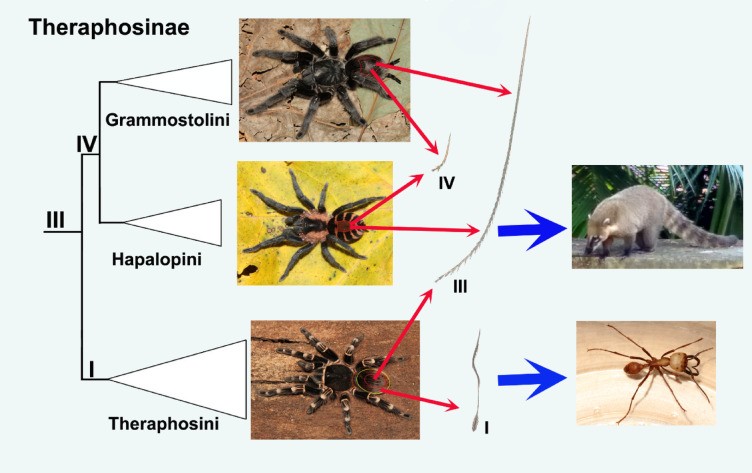
Simplified cladogram of Theraphosinae based on Turner et al.^30^, showing the distribution of morphological types of urticating setae, their distributions on the abdomen of spiders and their use against predator targets. Type III is a putative synapomorphy of Theraphosinae, with some reversals in species of Grammostolini, Hapalopini and Theraphosini. Type IV may be a synapomorphy of Grammostolini + Hapalopini, but with reversals and parallelisms. Type I is a synapomorphy of Theraphosini, and only a few reversals are known. The relatively small areas on the abdomen of spiders, indicated in red, have urticating setae of types III or III and IV, which are located mainly in the center of the abdomen (see also Fig. 2 for a more detailed view). In Theraphosini, the area with type III setae is surrounded by a large area with type I setae, indicated in yellow. Thus, most of the dorsal region of the abdomen of Theraphosini species is covered with urticating setae. While all theraphosine species possess type III urticating setae, which can be used against vertebrates, only theraphosini species bear type I setae, which can be used against ants.

Biswas et al.^18^ tested the asymmetry of species richness distribution among theraphosid subfamilies, concluding that clade age is insufficient to explain the high diversity of Theraphosinae. They concluded that the species richness of Theraphosinae could be attributed to the presence of urticating setae in this clade, which may have reduced the extinction rate^18^. However, the role of specific types of urticating setae was not considered in their study. While type III urticating setae, used against vertebrates, are widely distributed in Theraphosinae and their action is well understood, type I urticating setae, which are morphologically complex, have a poorly understood function. Type I urticating setae are weakly urticating to vertebrates, are distributed on the sides of the spider’s abdomen, which is not the primary area touched by the spider in defensives displays (Fig. 2C-D), and are not easily transported by air. However, they are present in an important clade of Theraphosinae, the tribe Theraphosini, which contains 53.34% of its species (Fig. 8).

Theraphosinae and Neotropical army ants likely originated after the separation of South America from Africa, approximately 50 and 37 million years ago, respectively^18,29^. Neotropical army ants appear to have arisen in tropical South America, diversified during a long period of isolation, and did not disperse to North America until the late Tertiary^21^. The species belonging to the clade of theraphosine tribes Grammostolini and Hapalopini lack type I urticating setae, which appear in the sister clade Theraphosini^30^ (Fig. 8). Both ancestors, prey and predator, have shared the same geographic region in South America for millions of years. The emergence of type I urticating setae, while offering protection against ant predation, may have allowed a significant portion of New World tarantulas to conquer extensive areas, explore new habitats, and live longer, reaching larger sizes. This could be a working hypothesis to be tested in future research to determine whether ant predation plays a role in the evolutionary pressure driving the evolution of tarantulas, especially those from the Americas and the Caribbean, which have the greatest diversity of tarantulas and the largest spider species in the world.

## Methods

### Specimens tested

Two specimens of tarantulas (Theraphosidae, Theraphosinae, Theraphosini) of the species *Acanthoscurria geniculata*, four specimens of *Lasiodora parahybana*, and a single specimen of *Lasiodora klugi* and *Lasiodora benedeni*, all females, were used to test the spiders’ defensive behavior when in contact with two species of ants, *Eciton burchelli* and *Camponotus rufipes* (Table 2). These theraphosid species from the Brazilian Amazon and the Brazilian Atlantic Forest live inside burrows dug into the soil, under fallen tree trunks or rocks, and are sedentary sit-and-wait nocturnal predators. They are representatives of the theraphosid fauna of Brazil, comprising two genera and four species from seven localities and two biomes. These are also large species that possess two types of urticating setae, types I and III.

*Eciton burchelli* (Formicidae, Dorylinae) is a large ant species that is 3 to 12 mm in length. They are well-known army ants that can be found exclusively in tropical habitats from Mexico to Argentina^20,21^. These ants are voracious generalist predators that form swarms that can contain up to 2 million workers and forage in the leaf litter of forests^21^. *Camponotus rufipes* (Formicidae, Formicinae) is a large ant species that is 6–15 mm in length. The species is widely distributed in the Neotropical region^31^, both in savannah and in the rainforest^32^. These omnivorous ants feed on fruits on the ground, plant exudates, and insect prey^32^. *Eciton* was tested due to its well known predatory behavior against a wide range of prey. *Camponotus* was included to test whether the spider’s observed behavior against *Eciton* could be species-specific. Therefore, another ant of a similar size that also feeds on invertebrates, but is not closely related, was chosen. *Camponotus* was also tested in a previous article on theraphosid molting webs with incorporated urticating setae^15^. Both ant species have a geographic distribution similar to that of the tarantula species tested.

All tarantulas, except one, were collected in the field and kept in the bioterium of the Laboratório de Ecologia e Evolução, Instituto Butantan. *Eciton burchelli* are from Mairiporã, state of São Paulo, Brazil, and *Camponotus rufipes* are from São Paulo, state of São Paulo, Brazil.

### Behavioral observations

In the first experiment, which was carried out in 2004, the specimens Ag1 and Lb were transferred from their cages to a plastic cage or an acrylic box. After five minutes, ten *Eciton burchelli* ants were released into the box. In a second experiment, which was carried out in 2025, the specimens Ag2, Lk, Lp1 and Lp2 were transferred from their cages to a plastic tray. After five minutes, ten *Eciton burchelli* ants from Mairiporã, state São Paulo, Brazil, were released into the plastic tray. Another experiment testing the omnivorous ant *Camponotus rufipes* was carried out in 2025 via the same methodology as that used for *Eciton burchelli* and the same spiders, Ag1, Lk, Lp1, Lp2, Lp3 and Lp4. If, after fifteen minutes of releasing the ants, no interaction between the spider and the ants was observed, the experiment was ended and considered negative. When there was interaction, the experiment continued for at least another fifteen minutes to record possible additional interactions.

The behavioral repertoire observed in tarantulas and ants is presented in Table 4.

**Table 4:** Behavioral repertoire observed in tarantulas and ants.

Behavior	Definition
*Tarantulas*	
Moving	Tarantula moves its legs or walks
Abdomen raising	Tarantula makes an upward abdominal movement
Abdomen tilting	Tarantula tilts its abdomen toward an approaching ant
Silk raising	Tarantula raises its abdomen, causing the silk threads attached to the spinnerets to rise. The ants trapped in the threads end up being lifted along with it
Spinning	Tarantula moves its spinnerets or walks, leaving silk threads attached to the ground
Abdomen rubbing, left leg	Tarantula raises its left leg, touches the lower left posterolateral region of its abdomen with its metatarsus, and quickly lowers the leg, rubbing its abdomen and releasing urticating setae. The leg moves away from the abddomen and is raised again, restarting the cycle. These movements are rapid, roughtly 3 to 5 per second.
Abdomen rubbing, right leg	As above, but with the right leg.
Body rotation	Tarantula moves around its axis, spinning towards an approaching ant
*Ants*	
Moving	Ant moves through the space. Due to the number of ants, it was considered that they are in constant motion, except when trapped by silk threads with urticating setae or exhibiting other behaviors after such contact
Touching the tarantula	Ant comes into contact with any part of tarantula’s body, causing a reaction or not
Entangled to another ant	Ant contamined with urticating setae comes into contact with another ant, and the two become entangled
Trapped in the web	Ant comes into contact with silk threads that have urticating setae and gets trapped in the web
Struggling	Ant trapped in a web with urticating setae struggles unsuccessfully to free itself

### Equipment

The behaviors of the spiders and ants were recorded with a Sony 8 mm recorder (2004) or with a Canon T3i camera (2025). Photos were taken with a Canon T3i or Canon EOS Rebel camera. The recorded videos were examined using the Behavioral Observation Research Interactive Software (BORIS), from which an ethogram was obtained^33^.

Photomicrographs were obtained with a QUANTA 250 scanning electron microscope (FEI Company) from the Laboratório de Biologia Celular, Instituto Butantan, or with a Leica DM2500 microscope with a DFC 450 camera attached, combined with Leica LAS Montage and LAS 3D modules.

## Supplementary Information

Below is the link to the electronic supplementary material.

Supplementary Material 1

## Data Availability

All data generated or analysed during this study are included in this published article and its Supplementary Information files.

## Declarations

### Approval for animal experiments

Under Brazilian law, studies involving invertebrates are exempt from ethical committee review.
